# Melatonin improves fertilization rate in assisted reproduction: Systematic review and meta-analysis

**DOI:** 10.1016/j.clinsp.2024.100397

**Published:** 2024-07-05

**Authors:** Eduardo Carvalho de Arruda Veiga, Marise Samama, Fabio Ikeda, Giovanna Santos Cavalcanti, Amanda Sartor, Suelen Fernanda Parames, Edmund C. Baracat, Joji Ueno, Jose Maria Soares Junior

**Affiliations:** a GERA Instituto de Ensino e Pesquisa em Medicina Reprodutiva de, São Paulo, SP, Brazil; b Department of Obstetrics and Gynecology, Hospital das Clínicas, Faculdade de Medicina de Ribeirão Preto da Universidade de São Paulo (FMRP-USP), São Paulo, SP, Brazil; c Department of Gynecology, Escola Paulista de Medicina, Universidade Federal de São Paulo (EPM/UNIFESP), São Paulo, SP, Brazil; d Lim-58 - Laboratório de Ginecologia Estrutural e Molecular da Disciplina de Ginecologia, Departamento de Obstetrícia e Ginecologia, Hospital das Clínicas, Faculdade de Medicina da Universidade de São Paulo (HCFMUSP), São Paulo, SP, Brazil

**Keywords:** Melatonin, Ovarian hyperstimulation syndrome, Assisted reproduction outcomes, In vitro fertilization, Human reproduction, Sytematic review

## Abstract

**Background:**

Melatonin is a hormone produced by the pineal gland and it has antioxidant properties.

**Aim:**

This study aimed to evaluate the effects of melatonin on assisted reproductive technologies through a systematic review and a meta-analysis.

**Materials and methods:**

Search strategies were used in PubMed and in other databases covering the last 15 years. After screening for eligibility, 17 articles were selected for the systematic review. For the meta-analysis statistics, two groups were formed, the treatment group (with melatonin) and the control group (without melatonin) for various assisted reproduction outcomes.

**Results:**

The main results were that no statistical differences were found concerning the clinical pregnancy outcome (*p* = 0.64), but there was a statistical difference with respect to Mature Oocytes (MII) (*p* = 0.001), antral follicle count (*p* = 0.0002), and the fertilization rate (*p* ≤ 0.0001).

**Conclusions:**

Melatonin had beneficial effects such as the improvement in the fertilization rate, although the authors did not obtain significance in the clinical pregnancy rate.

## Highlights

• Melatonin had beneficial effects on outcomes in assisted reproductive technologies, but it had no influence on pregnancy.
• Melatonin had beneficial effects such as the improvement in the fertilization rate.
• Melatonin has a beneficial effect on the formation of mature oocytes (MII).

## Introduction

Infertility affects millions of women worldwide and is one of the main causes of the unfulfilled dream of having a child.^1^ Assisted reproduction techniques may be accompanied by complications such as Ovarian Hyperstimulation Syndrome (OHSS), which can be fatal.^2^ As many as 20 % to 33 % of In Vitro Fertilization (IVF) cycles are affected by mild OHSS, whereas moderate to severe OHSS reportedly occurs in 3 % to 8 % of patients.^3^

Melatonin is a hormone that is primarily produced by the pineal gland. Two characteristics worth emphasizing are its antioxidant properties and its capacity to improve mitochondrial functions in female germ cells, which can lead to benefits in human reproduction treatments.^4^, ^5^, ^6^ Melatonin plays a role in sleep and in physiological oocyte maturation.^7^ Both in vitro and in vivo studies, in animals and in humans, show the benefits of melatonin as a substance that reduces the oxidative stress of cells related to reproduction, even improving fertilization rates.^8^

Two recent works have demonstrated that melatonin not only alleviates reactive oxygen species, but also improves apoptosis and that it may clinically benefit women who have developed OHSS.^9^^,^^10^ Other works discuss the important role, still under study, of melatonin related to steroid sex hormones, in one of these works it was observed that melatonin plays a role in maintaining follicular function through the production of progesterone,^11^ while in the most recent review, it is considered that melatonin plays a role not only in the production of progesterone but also in estrogen and spermatogenesis.^12^ Among the positive results of using melatonin for women on Assisted Reproductive Technology (ART) are increases in the number of mature oocytes, the fertilization rate, the number of high-quality embryos, and, in some cases, increased pregnancy rates.^13^^,^^14^

Given the above, this study aimed to evaluate the effects of melatonin on assisted reproductive technologies through a systematic review and a meta-analysis.

## Materials and methods

For the systematic review, the authors drew on several articles and guidelines, including Berstock et al., 2019,^15^ Hennessey et al., 2019,^16^ and Page et al., 2021.^17^ The meta-analysis was conducted in accordance with Higgins et al., 2022.^18^

### Search strategy

**Fig. 1:**
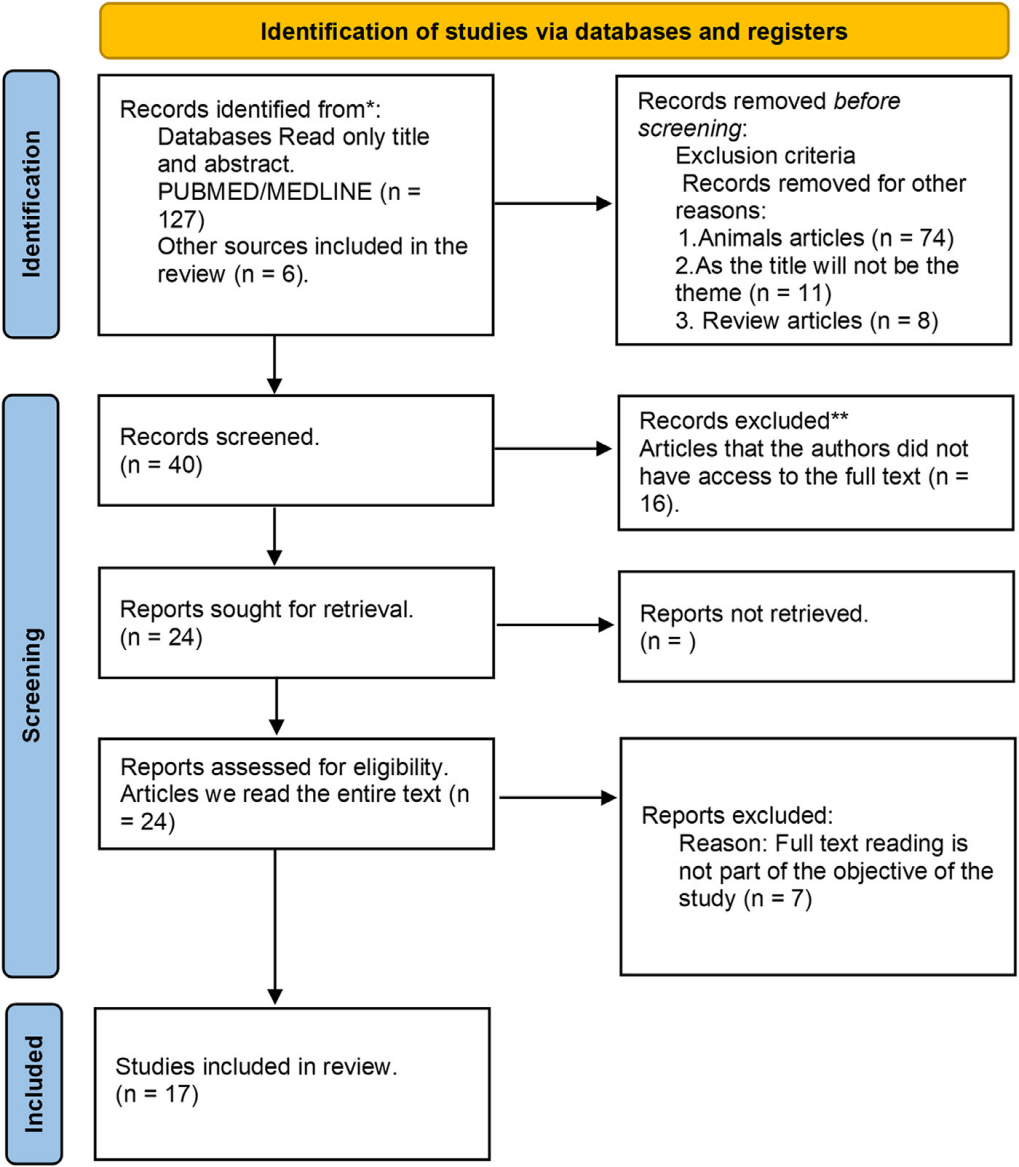
Flowchart of the systematic review. * Consider, if feasible to do so, reporting the number of records identified from each database or register searched (rather than the total number across all databases/registers). ** If automation tools were used, indicate how many records were excluded by a human and how many were excluded by automation tools. From: Page MJ, McKenzie JE, Bossuyt PM, Boutron I, Hoffmann TC, Mulrow CD, et al. The PRISMA 2020 statement: an updated guideline for reporting systematic reviews. BMJ 2021;372:n71. doi:10.1136/bmj.n71. For more information, visit: http://www.prisma-statement.org/.

The studies selected for this review were published between January 2008 and April 2023, and they are indexed in PubMed and Scielo (Fig. 1). The initial search yielded 127 articles. After applying the eligibility criteria, they were narrowed down to the 17 articles included in this systematic review. Fig. 1 shows the selection process in detail.

Drawing on Page et al., 2020,^17^ the P (Population) in the P.I.C.O. of this systematic review is patients, i.e., the female research participants who were selected to take exogenous melatonin and who had or had not ovarian hyperstimulation syndrome, depending on the article; the I (Intervention) is the administration of exogenous melatonin; the C (Comparison) is the comparison of the control group and the experimental group (exposed to melatonin); the O (Outcomes) is the outcomes as described in Table 1.

**Table 1:** Exogenous melatonin in previous clinical studies.

Authors	Years	Study design	Technique	Melatonin treatment	Justifications for inclusion criteria	Main outcomes
Tamura et al. [11]	2008	Controlled Clinical Trial	IVF ET	3 mg/day	The study has results that melatonin improves among other aspects of ARTs and fertility rates	improves oocyte and embryo quality and better fertility taxes.
Unfer et al. [19]	2011	Clinical Trial	IVF	3 mg/day	The study has results that melatonin improves oocyte quality	had improved on pregnancy rate
Batioglu et al. [18]	2012	Randomized Controlled Trial	IVF ET	3 mg/day	The justification for the inclusion is the efficacy of melatonin administered in improving oocyte quality	improves oocyte and embryo quality
Fernando et al. [17]	2014	Clinical Trial	IVF ICSI	4 mg/twice per day	Double-blind randomized study evaluating melatonin in infertility treatments	Melatonin in ART will be the first trial designed to determine a relationship of melatonin on clinical pregnancy rates.
Nishihara et al. [20]	2014	Clinical Trial	IVF	3 mg/day	The justification for the inclusion is the efficacy of melatonin administered in improving oocyte quality	improves oocyte and embryo quality
Jahromi et al. [21]	2017	Randomized Controlled Trial	IVF	3 mg/day	Double-blind randomized study evaluating melatonin in infertility treatments in women with low ovarian reserve	improves oocyte and embryo quality
Tong et al. [22]	2017	Clinical Trial	IVF ICSI	Melatonin measument in folicular fluid with range was 2.3‒1000 pg/mL.	The justification for the inclusion is that melatonin levels can be markers and predictors of low ovarian reserve and better results in IVF	improves oocyte and embryo quality
Zheng et al. [23]	2017	Clinical Trial	IVF	Melatonin measument in folicular fluid.	The work seals the concentrations of melatonin in the follicular fluids and their role in human reproduction	We have demonstrated that higher folicullar fluid melatonin concentrations were related to better ART outcomes
Ma et al. [24]	2018	Clinical Trial	IVF ICSI ET		This study was the only one that did not have the presence of melatonin, therefore it was important for the inclusion criteria in the present systematic review because it worked with studies of two fluids of two follicular follicles in women with ovarian hyperstimulation syndrome.	Follicle count measured on the day of hCG administration was the only predictive factor for the occurrence of OHSS
Espino et al. [25]	2019	Clinical Trial	IVF	3 mg/day or 6 mg/day	The authors studied the use of melatonin in infertilities with no apparent or apparent cause in unexplained infertilities	improves oocyte and embryo quality
Fernando et al. [26]	2019	Randomized Controlled Trial	IVF	2, 4 or 8 mg/twice a day	This work, despite having arguments that go against current literature, is important for studying melatonin in ovarian vascular indices	Melatonin and vascular indices cannot predict the number or quality of oocytes or embryos obtained in an IVF cycle.
Li et al. [27]	2019	Case control Study	IVF ICSI ET	Melatonin measument in folicular fluid.	The authors studied in the same work the functions of melatonin in ovarian hyperstimulation syndrome	Role of melatonin as a predictor of ovarian hyperstimulation syndrome
Zheng et al. [28]	2019	Clinical Trial	IVF	Melatonin measument in folicular fluid	This work was important to be selected because it was the first to demonstrate that melatonin in the follicular fluids is significantly increased in women with OHSS	The authors studied in the same work the functions of melatonin in ovarian hyperstimulation syndrome
Espinola et al. [29]	2020	Prospective Randomized and Controlled Pilot Study	IVF	1 mg/day	As justifications for the inclusion of this work, there is a randomized study that studied vitamin D, melatonin, or myo-inositol and folic acid in assisted reproduction	The main failure was that increased vitamin D levels were positively correlated with IVF implantation rates.
Wdowiak et al. [30]	2020	Prospective Randomized and Controlled Trial	IVF ICSI	1 mg/day	A study comparing other two substances together with melatonin in women with OHSS	A combination of myo-inositol, vitamin D and melatonin including better fertilization and pregnancy outcomes as well as reduced risk of OHSS
Li et al. [31]	2021	Randomized Controlled Trial	IVF IVM	MT in fluid folicular in IVF and addition in vitro culture of 10^−5^ mol/L melatonina in IVM.	It was a pilot study that compared IVF protocols with in vitro maturation and the presence of melatonin and its results in human reproduction.	Melatonin supplementation has efficacy in clinical results of assisted reproduction as higher rates of oocytes in IVF
Zheng et al. [32]	2022	Clinical Trial	‒	Melatonin treatment in 10 µM cell culture	The authors studies the functions of melatonin in ovarian hyperstimulation syndrome	Melatonin attenuated reactive oxygen species during apoptosis

IVF, In Vitro Fertilization; ICSI, Intracytoplasmic Sperm Injection; IVM, In Vitro Maturation; MT, Melatonin; OHSS, Ovarian Hyperstimulation Syndrome.

This review was conducted following the PRISMA (Preferred Reporting Items for Systematic Reviews and Meta-Analysis) recommendations.^15^

### Inclusion and exclusion criteria

The exclusion criteria covered animal studies (*n* = 74), articles whose titles or abstracts did not fall within the scope of the present study (*n* = 11), and review articles (*n* = 8). An additional 16 articles were excluded for lack of access to the full text because they were not freely accessible. The remaining 24 articles were fully read. By applying a second round of exclusion criteria, 7 more studies were screened out. The 17 articles that remained met the inclusion criteria of this study (see Table 1 for details) and were thus included in the study (Fig. 1). The inclusion and exclusion criteria were based on Page et al., 2020.^17^

### Statistical analysis

For descriptive analysis, calculations were made for means, standard deviations, mean differences, and odd ratios with a 95 % Confidence Interval. Meta-analysis was carried out with the Review Manager 5.4.1 software program (Cochrane Collaboration, Oxford, UK). For the 95 % CI and the overall effect size, values of *p* ≤ 0.05 were assumed for significant differences.

## Results

Melatonin is involved in a number of the body's physiological processes, one of which is regulating fertility. The articles included in this study are detailed in Table 1, which also includes information about the authors, publication year, methods, melatonin treatment, rationale for article inclusion, and key results or outcomes. A significant finding of this systematic review was that melatonin plays a role in the improvement of oocyte and embryo quality.^19^, ^20^, ^21^, ^22^, ^23^, ^24^, ^25^, ^26^, ^27^, ^28^, ^32^, ^33^, ^34^

### Meta-analysis of assisted reproduction outcome variables

**Fig. 2:**
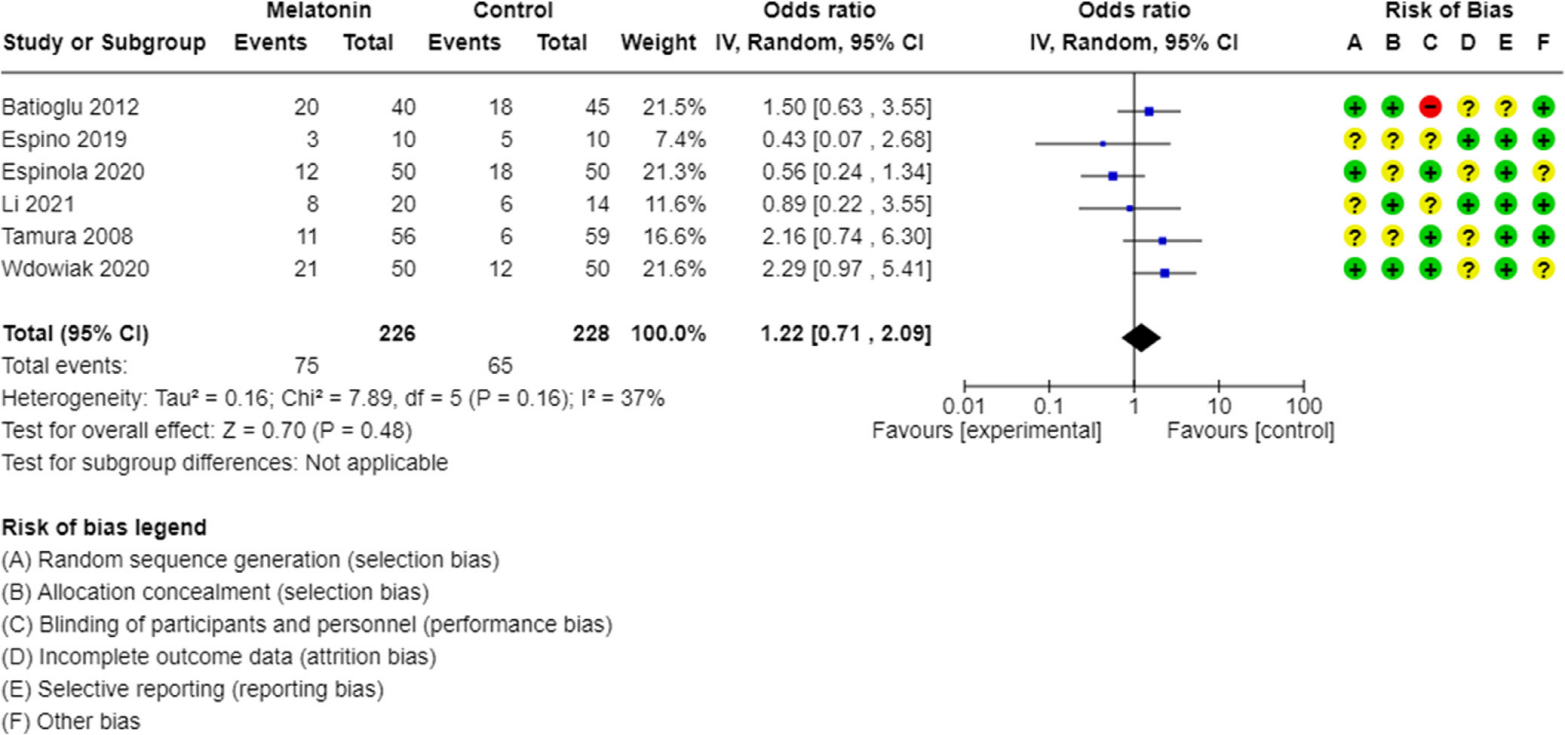
Meta-analysis of clinical pregnancy rate in patients receiving and not receiving melatonin.

The comparison between melatonin intake and the clinical pregnancy rate of assisted reproduction yielded no statistical difference. Only 6 studies included this variable and with the following results: *p* = 0.64, I^2^ = 37 % (Fig. 2); risk ratio of 1.22 (0.71‒2.09). Caution is needed in interpreting this outcome, for there are numerous other variables involved in a healthy pregnancy resulting from assisted reproduction techniques, including the physiological conditions necessary for achieving pregnancy.

**Fig. 3:**
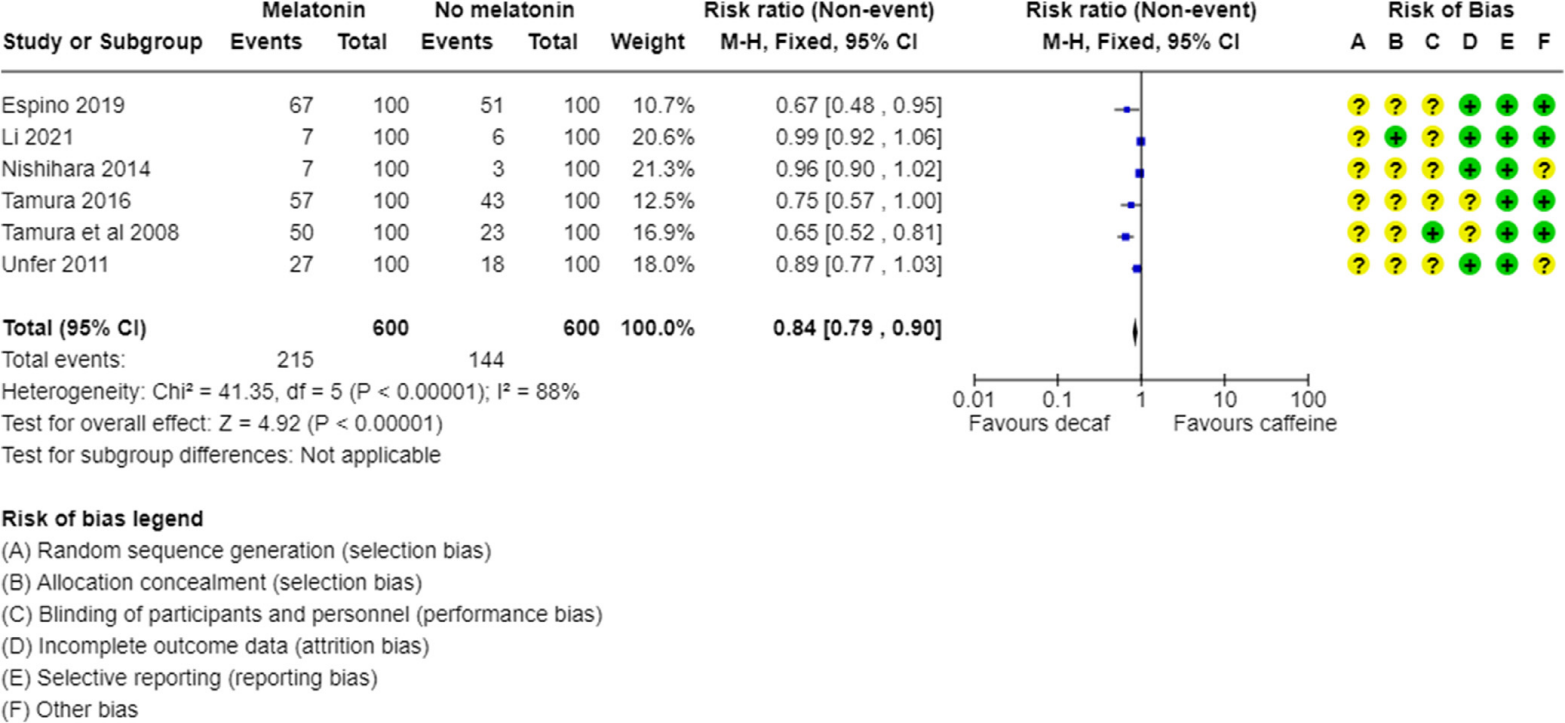
Meta-analysis of fertilization rate in patients receiving and not receiving melatonin.

The meta-analysis of the fertilization rate outcome in percentage (%) showed a positive effect of the melatonin treatment, as the difference between the melatonin treatment groups and the control groups was statistically significant (*p* ≤ 0.00001, I^2^ = 88 %, Fig. 3; risk ratio = 0.84 [0.79, 0.90]). The fertilization rate is an important indicator of reproductive outcomes, and the group of women who took melatonin had improved results compared to the group of women who did not take it (Fig. 3).

In the only four studies addressing follicle count, melatonin had positive effects on the growth of follicles as shown by *p* ≤ 0.00001, I^2^ = 85 % (Fig S2), which points to statistical significance.

Five studies measured the Mature oocyte (MII). Statistically significant values were found, and they are *p* = 0.001, I^2^ = 87 % (Fig. S1). The MII oocyte is the female germ cell in an ideal state of maturation for fertilization.

A meta-analysis of maternal age in years was performed in 11 studies, and no statistically significant difference was found (*p* = 0.64 and I^2^ = 64 %). Body mass index (kg/m^2^) was a sociodemographic variable analyzed in 8 studies, with no significant differences in outcome (*p* = 0.59, I^2^ = 77 %). A third sociodemographic variable was women's infertility time, and it approached the significance level at best (*p* = 0.06, I^2^ = 0 %).

In short, melatonin had beneficial effects as shown by the increased fertilization rate and other outcomes of the reproductive process. The clinical pregnancy rates, however, were not significantly different in the group comparison.

## Discussion

The main findings were that women who took melatonin had an improved fertilization rate and reaped other benefits from assisted reproductive technologies. However, melatonin intake did not result in a higher clinical pregnancy rate.

Of the 17 studies selected for this systematic review, only two articles, those by Li et al., 2019^35^ and Zhang et al., 2023,^34^ deal directly with the subject of ovarian hyperstimulation and the way in which melatonin can be a hormone that aids reproductive results. Li et al., 2019, concluded that melatonin produced by the follicular follicle helps predict OHSS, while Zhang et al., 2020, took a deeper approach. They demonstrated how melatonin, in addition to having anti-apoptotic properties, can improve oxidative stress in OHSS and concluded that it can indeed prevent OHSS. In a recent study by Hu et al., 2020,^36^ promising results were obtained, but they differ from those of this meta-analysis, in which the clinical pregnancy rate improved with the administration of melatonin to patients with an OR of 1.43. However, their study comprised only articles with an RTC design, an advantage offset mainly by the low-quality bias and the heterogeneity of the articles. In a recent meta-analysis by Mejihede et al. 2021^35^ with 7 articles from randomized controlled trials, oral melatonin supplementation resulted in an increase in the number of mature oocytes, and a trend for increasing CPR, albeit not significant.^32^ A recent study also verified the relationship between melatonin and the increase in gene expression in rats of follistatin and of Inhibin Beta-A, substances necessary for good hormonal regulation of the ovary and oocyte maturation.^35^ One of the mechanisms may be through the melatonin receptor in ovarian follicles, but there are also other mechanisms that do not depend on the receptor and are related to antioxidant substances.^32^, ^34^, ^35^, ^37^

Some recent studies demonstrate that melatonin either from follicular fluid, granulosa cells, or exogenous sources has important roles concerning the quality of oocytes. It can delay the aging of the ovaries and their functions and improve the antioxidant properties of the oocytes, leading to improved reproductive outcomes such as an improved fertilization rate.^5^^,^^28^, ^32^, ^34^, ^35^

The strength of the present work lies in demonstrating through meta-analysis that variables analyzed at the onset of assisted reproduction techniques improved to benefit the women who used exogenous melatonin. On the other hand, in the author's judgment, an important limitation is that in nearly half of the studies, the risk of bias was unclear or was not mentioned, impairing the quality of the studies. Another limitation, and the most important one, is that there was no difference in the clinical pregnancy rate between the groups. Further, there were not enough studies among the selected articles to analyze the main variable of assisted reproduction, namely the rate of live births.

### Limitations of the study

This study has two main limitations. First, it is a systematic review and as such there is no data collection. Also, the results are those of previously published articles. Second, with respect to the meta-analysis of the clinical rate of pregnancy variable, there is no statistical difference between the use and the nonuse of melatonin, despite its beneficial molecular and cellular effects as judged by the values presented in the articles.

## Conclusion and future perspectives

Melatonin is not a substance that has been used frequently in assisted reproduction. However, it has the following advantages for use in clinical practice: it is low cost; it is commercially available; it is a hormone produced by our own body and thus has no side effects; it has, as one of its main physiological actions, the capacity to reduce the oxidative stress of oocytes, but due to the few existing studies, this feature is still being overshadowed by the main results of human reproduction, such as clinical pregnancy rate and live birth rate. Therefore, the authors suggest that this line of research into melatonin use in assisted reproductive technologies be expanded with double-blind randomized multicenter studies. Melatonin had beneficial effects such as the improvement in the fertilization rate, although the authors did not obtain significance in the clinical pregnancy rate.

Additional studies, such as double-blind randomized clinical trials with many participants, are needed, particularly as regards melatonin action on ovarian hyperstimulation syndrome.

## Funding

No funding.

## Declaration of competing interest

The authors declare no conflicts of interest.

## Appendix Supplementary materials

Image, application 1
