# Supplementary material for: Melatonin improves fertilization rate in assisted reproduction: Systematic review and meta-analysis
Source: Clinics (Sao Paulo). 2024 Jul 5;79:100397. doi: 10.1016/j.clinsp.2024.100397 (PMC11265587; doi:10.1016/j.clinsp.2024.100397)

**CLINICS-D-24-00071_Supplementary Material**

**Figure S1** Meta-analysis of Mature oocyte outcome (MII) in patients receiving and not receiving melatonin.


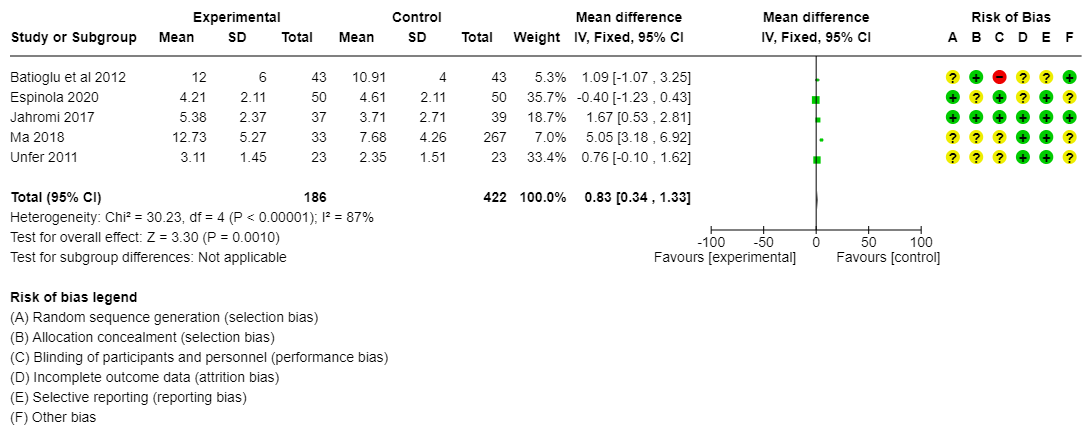


**Figure S2** Meta-analysis of antral follicle count in patients receiving and not receiving melatonin.


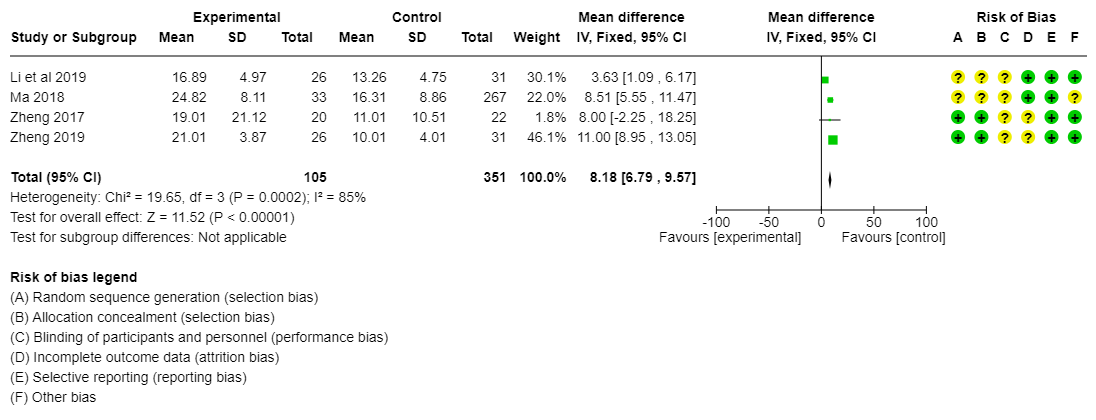

Supplement: Supplementary file 1 [file mmc1.docx]
